# A statistical mechanics model for determining the length distribution of actin filaments under cellular tensional homeostasis

**DOI:** 10.1038/s41598-022-18833-1

**Published:** 2022-08-24

**Authors:** Yuika Ueda, Daiki Matsunaga, Shinji Deguchi

**Affiliations:** Division of Bioengineering, Graduate School of Engineering Science, Osaka University, 1-3 Machikaneyama, Toyonaka, Osaka 560-8531 Japan

**Keywords:** Biophysics, Cell biology, Cytoskeleton, Biological physics

## Abstract

Tensional homeostasis is a cellular process whereby nonmuscle cells such as fibroblasts keep a constant level of intracellular tension and signaling activities. Cells are allowed thanks to tensional homeostasis to adapt to mechanical stress, but the detailed mechanism remains unclear. Here we address from a theoretical point of view what is required for maintaining cellular tensional homeostasis. A constrained optimization problem is formulated to analytically determine the probability function of the length of individual actin filaments (AFs) responsible for sustaining cellular tension. An objective function composed of two entropic quantities measuring the extent of formation and dispersion of AFs within cells is optimized under two constraint functions dictating a constant amount of actin molecules and tension that are arguably the two most salient features of tensional homeostasis. We then derive a specific probability function of AFs that is qualitatively consistent with previous experimental observations, in which short AF populations preferably appear. Regarding the underlying mechanism, our analyses suggest that the constraint for keeping the constant tension level makes long AF populations smaller in number because long AFs have a higher chance to be involved in bearing larger forces. The specific length distribution of AFs is thus required for achieving the constrained objectives, by which individual cells are endowed with the ability to stably maintain a homeostatic tension throughout the cell, thereby potentially allowing cells to locally detect deviation in the tension, keep resulting biological functions, and hence enable subsequent adaptation to mechanical stress. Although minimal essential factors are included given the actual complexity of cells, our approach would provide a theoretical basis for understanding complicated homeostatic and adaptive behavior of the cell.

## Introduction

Homeostasis is responsible for the maintenance of living systems, in which their internal and external elements exhibit complex interactions. In addition to the well-known presence at the organismal level, homeostasis has actually been recognized to be of importance at the individual cellular level. Tensional homeostasis is one of the known homeostatic mechanisms, by which nonmuscle cells such as fibroblasts, endothelial cells, and cancer cells are allowed to sustain a constant level of tension through turnover of their constituent proteins^1–4^. Cellular tension is thus relaxed to the basal level over time even upon mechanical stress that disturbs intracellular tensional balance^5,6^. Mechanical stresses such as cell stretch and fluid shear stress are known to trigger signaling pathways, and importantly such signals, typically associated with cell growth and inflammation, are suppressed concomitantly with the recovery of cellular tension. Thus, tensional homeostasis plays crucial roles in functional adaptation of nonmuscle cells to mechanical environment and consequently has been implicated in many diseases such as atherosclerosis^7–12^; however, the detailed mechanism remains largely unknown.

Given that living systems are comprised of many structural/functional “layers” that are highly complicated but closely associated with each other, deciphering the homeostasis at the cell layer level will contribute to deeper understanding of the whole homeostasis and resulting adaptive behavior at the organismal level. With this motivation, we must be in a position to tackle this challenging but exciting topic, namely the mechanism underlying cellular tensional homeostasis. Here we discuss physical aspects of the mechanism from a theoretical point of view bridging subcellular and cellular layers. Specifically, we address what is the requirement for actin filaments (AFs), a major cytoskeletal component that undergoes turnover, to allow for sustaining a constant level of tension over the cytoplasm. A constrained optimization problem is then formulated to describe the probability function of the length of individual AFs. Our result yielded a probability function with no local maxima unlike the Gaussian distribution but consistent with experimental observations, describing how the homeostasis at the cellular level is achieved on the underlying basis of the molecular level.

## Methods

### Constraint function

We aim to derive the length distribution of the individual AFs in cells according to the method of Lagrange multipliers. The number of actin monomers that constitute an AF with a length of $l_{{\text{i}}}$ is $n_{{\text{i}}}$ where ${\text{i}}$ is a positive integer. We normalize the length (effective diameter) of individual actin monomers to be unity, and AF length is proportional to the number of constituent actin monomers; accordingly, $l_{{{\text{i}} + 1}} = l_{{\text{i}}} + l_{1} = l_{{\text{i}}} + 1$. We consider two equality constraints. First, we assume that the total number of actin monomers within the cell, ${\text{N}}$, remains constant in accordance with the fact that actin is commonly used as a "housekeeping" protein in Western blotting analysis, and is thus described as

1 $$\begin{array}{*{20}c} {N = \sum n_{{\text{i}}} .} \\ \end{array}$$

The probability that actin monomers belong to a population of AFs with a length of $l_{{\text{i}}}$ is described by

2 $$\begin{array}{*{20}c} {p_{{\text{i}}} = \frac{{n_{{\text{i}}} }}{{\text{N}}}} \\ \end{array}$$

where the sum of the probabilities is unity given Eq. (1).

We impose another equality constraint to capture the essence of tensional homeostasis. Cells are known to sustain preexisting tension of a similar magnitude over the cytoplasm, which is borne by the meshwork of AFs undergoing interactions with myosin II^12–16^. These observations suggest that the expected value of the tension in individual AFs of the meshwork is constant. The expected value $F$ is then described as an equality constraint by

3 $$\begin{array}{*{20}c} {F = \sum p_{{\text{i}}} f_{{\text{i}}} } \\ \end{array}$$

where $f_{{\text{i}}}$ is a tension borne by an AF with a length of $l_{{\text{i}}}$. The tension in the AF meshwork, which finally gives rise to tensional homeostasis, is generated by the dynamic interaction with myosin II. To bear the tension, individual AFs must be long in length enough to make contact with other surrounding AFs to form a meshwork^17–20^. Otherwise, the tension is not transmitted across the cytoplasm even with the presence of myosin II activity. In other words, shorter AFs would have less opportunity to bear a tension within the meshwork compared to longer ones. Besides, because there is an upper limit of the myosin II activity, individual AFs would sustain up to a certain level of forces. In addition, while the level of myosin II activity differs along the length of stress fibers, that in AFs of the meshwork is typically in a similar state comprised mainly of mono-phosphorylated myosin regulatory light chain^21^. Therefore, the myosin II activity within the actin meshwork can be approximated to be spatially uniform. To capture these features, the relationship between $f_{{\text{i}}}$ and $l_{{\text{i}}}$ is described by a sigmoid function

4 $$\begin{array}{*{20}c} {f_{i} = \frac{{f_{0} }}{{1 + \exp \left( {l_{0} - l_{{\text{i}}} } \right)}}} \\ \end{array}$$

where $f_{0}$ and $l_{0}$ represent the maximum force and the length with the maximum slope of force, respectively (Supporting Material Fig. S1).

### Objective function

What is appropriate for the objective function of the constrained problem that characterizes tensional homeostasis? AFs, responsible for bearing cellular tension, are subject to turnover, by which the constituent monomers are polymerized and/or depolymerized. This continuous turnover allows intracellular AFs to fluctuate in length, and from an entropic perspective they are supposed to take as many states as possible at equilibrium. Thus, the probability distribution of AF length would be partly determined to increase a specific entropy that dictates formation of AFs with extensive fluctuations and is here described by

5 $$\begin{array}{*{20}c} {S_{{\text{f}}} = {\text{k}}_{{\text{b}}} lnW} \\ \end{array}$$

where ${\text{k}}_{{\text{b}}}$ and $W$ denote the Boltzmann constant and the number of states, respectively. Under the principle of equal a priori probabilities of each AF length, the number of states of actin molecules is described by

6 $$\begin{array}{*{20}c} {W = \frac{{{\text{N}}!}}{{n_{1} !n_{2} ! \cdots n_{{\text{m}}} !}}} \\ \end{array}$$

where ${\text{m}}$ is a group of AFs with the longest length. Using Stirling's approximation and Eqs. (1) and (2), Eq. (5) is rewritten as

7 $$\begin{array}{*{20}c} {S_{{\text{f}}} = - {\text{k}}_{{\text{b}}} N\sum p_{{\text{i}}} lnp_{{\text{i}}} } \\ \end{array}$$

(see Supporting Material for derivation). We adopt this formation-associated entropy as one of the objective functions.

As already discussed above, AFs must be distributed throughout the cytoplasm to form a meshwork where preexisting tension is sustained. As another objective function, we then consider an entropy related to the spatial distribution of AFs given that extensive dispersion of the AFs would allow the cells to be stabilized. Actin-based structures include directional architectures such as stress fibers that align in a specific direction, but the cytoplasmic “directionless” AF meshwork is more commonly present in nonmuscle cells^17–20^. The presence of the random meshwork is supposed to allow the cells to stabilize the whole cell architecture^13,14^ as well as to sense and respond to mechanical cues arising from arbitrary directions^11,22^. To evaluate this dispersion-associated entropy, we analyze the extent of intracellular regions where AFs are dispersed depending on their length; here, if all actin monomers form a single long AF, the region of the cell to be occupied, mechanically supported, and communicated by the AF will be limited in space; meanwhile, if actin monomers instead form many short AFs, they overall would be able to cover a large region upon dispersion.

**Figure 1:**
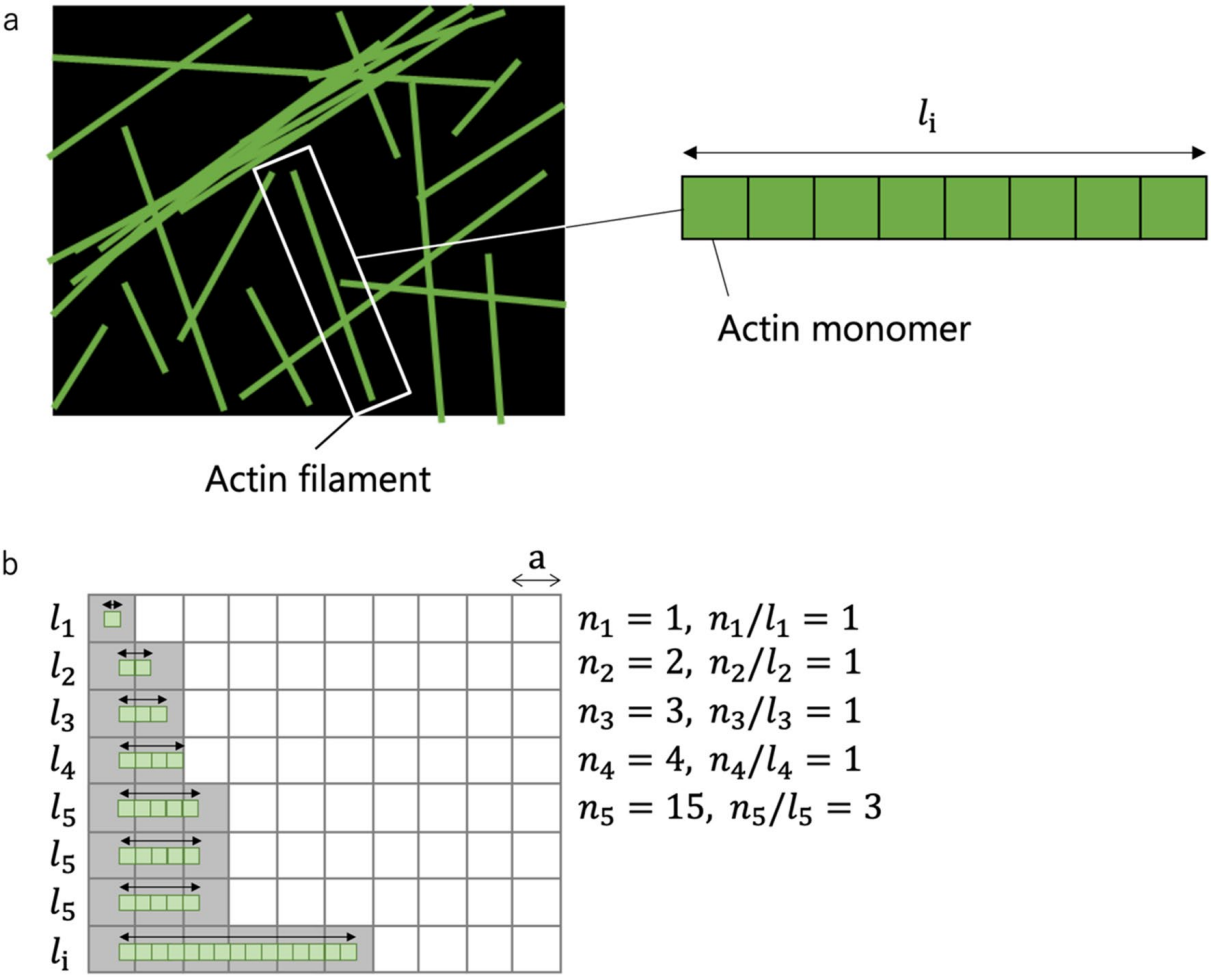
Schematic to explain $S_{d}$. (**a**) Schematic of the cytoplasm (black) where AFs (green) assumed to be formed by one-dimensional association of actin monomers to have a length of $l_{{\text{i}}}$ are spatially distributed. (**b**) The cytoplasm is divided by compartments with a length of ${\text{a}}$. AFs (green) occupy some of the compartments (gray). The arrows in the cell show the length of AFs. An example of $l_{{\text{i}}}$ comprising of 15 actin monomers is shown. See Supporting Material for detailed explanation.

For the analysis, the cytoplasm is modeled to consist of compartments, the number of which is sufficiently larger than that of AFs (Fig. 1). For each AF, the degree of intracellular dispersion is evaluated by counting the maximum number of compartments to which the AF can be distributed. The number of the compartments that an AF can occupy is partly determined by its length $l_{{\text{i}}}$ and the compartment size ${\text{a}}$. We do not consider deformation of AFs for simplicity, and thus only one-dimensional arrangement is analyzed. The maximum number of compartments that can be covered by all the existing AFs is expressed by the following equation (see Supporting Material for detailed explanation):

8 $$\begin{array}{*{20}c} {G = \frac{{n_{1} }}{{l_{1} }} + 2\mathop \sum \limits_{{{\text{i}} = 2}}^{{1 + {\text{a}}}} \frac{{n_{{\text{i}}} }}{{l_{{\text{i}}} }} + 3\mathop \sum \limits_{{{\text{j}} = 2 + {\text{a}}}}^{{1 + 2{\text{a}}}} \frac{{n_{{\text{i}}} }}{{l_{{\text{i}}} }} + \cdots = \frac{{n_{1} }}{{l_{1} }} + \mathop \sum \limits_{{{\text{j}} = 1}}^{{\frac{{{\text{m}} - 2}}{{\text{a}}} + 1}} \mathop \sum \limits_{{{\text{i}} = 2 + {\text{a}}\left( {{\text{j}} - 1} \right)}}^{{1 + {\text{aj}}}} \left( {{\text{j}} + 1} \right)\frac{{n_{{\text{i}}} }}{{l_{{\text{i}}} }}.} \\ \end{array}$$

As $n_{{\text{i}}} /l_{{\text{i}}}$ denotes the number of individual AFs of length $l_{{\text{i}}}$, we can compute using Eq. (8) the contribution of each length population to $G$, which we denote as $A_{{\text{i}}}$. In other words, Eq. (8) is alternatively described as

9 $$\begin{array}{*{20}c} {G = \sum A_{{\text{i}}} \frac{{n_{{\text{i}}} }}{{l_{{\text{i}}} }}.} \\ \end{array}$$

$G$ measures the extent of intracellular dispersion of AFs, which is identical in concept to entropy. To evaluate its effect on the stabilization of the cell, we introduce a constant ${\text{k}}_{{\text{b}}}^{^{\prime}}$ having the same unit as that of the Boltzmann constant ${\text{k}}_{{\text{b}}}$ to define dispersion entropy

10 $$\begin{array}{*{20}c} {S_{d} = {\text{k}}_{{\text{b}}}^{^{\prime}} \sum {\text{N}}\frac{{A_{{\text{i}}} }}{{l_{{\text{i}}} }}p_{{\text{i}}} .} \\ \end{array}$$

Given that formation entropy $S_{{\text{f}}}$ and dispersion entropy $S_{{\text{d}}}$ are independent of each other, we describe the overall objective function or the overall entropy of the system $S$ as the sum of them, i.e.,

11 $$\begin{array}{*{20}c} {S = - {\text{k}}_{{\text{b}}} N\sum p_{{\text{i}}} lnp_{{\text{i}}} + {\text{k}}_{{\text{b}}}^{^{\prime}} \sum {\text{N}}\frac{{A_{{\text{i}}} }}{{l_{{\text{i}}} }}p_{{\text{i}}} .} \\ \end{array}$$

Note that to be exact the overall entropy characterizing highly complex systems like cells would contain numerous entropy-associated factors with a form of the product/sum of them. For example, the product of two hierarchical entropies may be needed to appropriately characterize the overall system if we look at the details of the larger factor. The entropy in such a complex system will thus be practically determined by the final coarseness of the model. The sum, on the other hand, describes independent factors; but, if the effect is small enough, its contribution would be ignored. In the present case, we have considered the two independent entropies, i.e., how AFs are creased ($S_{{\text{f}}}$) and how AFs are dispersed ($S_{{\text{d}}}$), both of which are supposed be the two most essential factors in the AF-based cellular tensional homeostasis; and, their relative contributions are evaluated by changing the ratio of effective Boltzmann constants ${\text{k}}_{{\text{b}}}$ and ${\text{k}}_{{\text{b}}}^{^{\prime}}$, each of which relates the number of their respective possible microstates to the macroscopic cell state.

### Lagrange multipliers

Introducing Lagrange multipliers ${\upalpha }$ and ${\upbeta }$ for the constraint function Eq. (1) (divided by ${\text{N}}$ and substituted to Eq. (2)) and Eq. (3), respectively, the partial derivative of the Lagrangian function

12 $$\begin{array}{*{20}c} {L = S - \alpha \left( {\sum p_{{\text{i}}} f_{{\text{i}}} - F} \right) - \beta \left( {\sum p_{{\text{i}}} - 1} \right)} \\ \end{array}$$

is taken with respect to $p_{i}$ to find a specific distribution of AFs that maximizes the objective function of Eq. (12) under the constraints. Thus, the probability function of AFs that stabilizes the cell system at equilibrium is obtained by the method of Lagrange multipliers together with thermodynamic considerations to be

13 $$\begin{array}{*{20}c} {p_{{\text{i}}} = \frac{{{\text{exp}}\left[ {\left( {{\text{k}}_{{\text{b}}}^{^{\prime}} \frac{{A_{{\text{i}}} }}{{l_{{\text{i}}} }} - \frac{{{\text{V}}\lambda }}{{2{\text{TN}}}}f_{{\text{i}}} } \right)/{\text{k}}_{{\text{b}}} } \right]}}{{\sum {\text{exp}}\left[ {\left( {{\text{k}}_{{\text{b}}}^{^{\prime}} \frac{{A_{{\text{i}}} }}{{l_{{\text{i}}} }} - \frac{{{\text{V}}\lambda }}{{2{\text{TN}}}}f_{{\text{i}}} } \right)/{\text{k}}_{{\text{b}}} } \right]}}} \\ \end{array}$$

where ${\text{V}}$, $\lambda$, and ${\text{T}}$ denote the volume, preexisting strain, and temperature of the cell, respectively (see Supporting Material for derivation).

### Parameters

The following parameters are used unless otherwise stated: ${\text{N}}$ = 1,000, ${\text{k}}_{{\text{b}}}$ = 1.38 × 10^–23^ J/K, ${\text{T}}$ = 309.5 K, ${\text{V}}$ = 6 × 10^–16^ m^3^, ${\uplambda }$ = 0.2, $l_{1}$ = 1, ${\text{a}}$ = 10, $f_{0}$ = 10^–12^ N, and $l_{0}$ = 80. Here, the values for ${\text{T}}$ and ${\text{V}}$ were chosen from a typical temperature and cell volume, respectively; the value for ${\text{N}}$ was chosen arbitrarily; the values for the spatial parameters ${\text{a}}$ and $l_{0}$ are arbitrarily chosen and are normalized by $l_{1}$; the value for ${\text{k}}_{{\text{b}}}$ is taken from the Boltzmann constant; the value for ${\uplambda }$ is chosen from in vivo data on vascular smooth muscle cells, endothelial cells, and osteoblastic cells, in which the common value of ~ 0.2 was observed across the different cell types^12–16^; and, the value for $f_{0}$ is chosen from in vitro data on purified single actin filaments^23^, respectively. Thus, these basic parameters are taken primarily from vascular cells, but the effect of changing the major parameters will be examined.

## Results

### The relative effect of the two entropies

**Figure 2:**
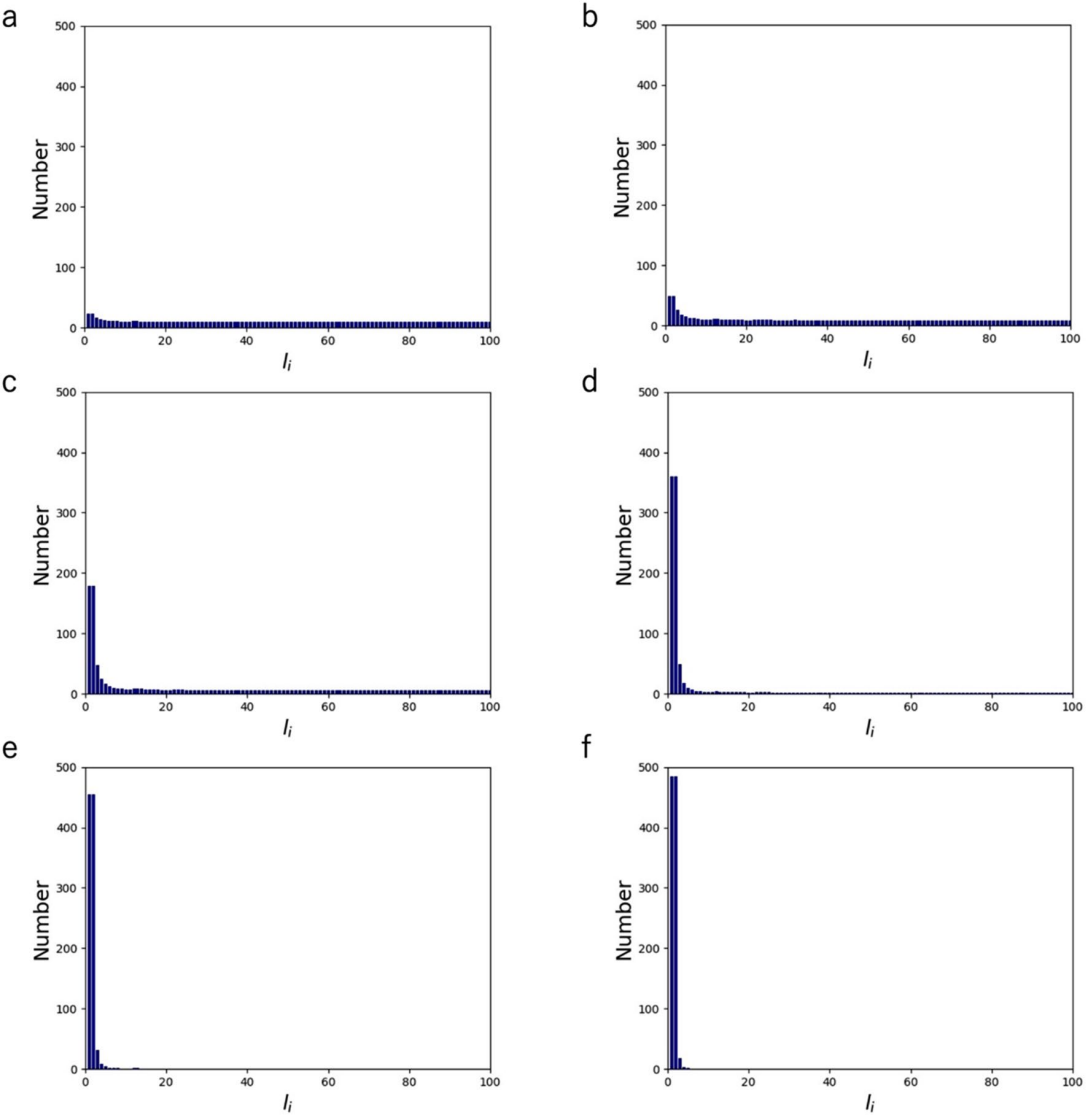
Length distribution of AFs comprising of ${\text{N}}$ = 1,000 monomers at ${\text{k}}_{{\text{b}}} ^{\prime}/{\text{k}}_{{\text{b}}}$ = 1 (**a**), ${\text{k}}_{{\text{b}}} ^{\prime}/{\text{k}}_{{\text{b}}}$ = 2 (**b**), ${\text{k}}_{{\text{b}}} ^{\prime}/{\text{k}}_{{\text{b}}}$ = 4 (**c**), ${\text{k}}_{{\text{b}}} ^{\prime}/{\text{k}}_{{\text{b}}}$ = 6 (**d**), ${\text{k}}_{{\text{b}}} ^{\prime}/{\text{k}}_{{\text{b}}}$ = 8 (**e**), and ${\text{k}}_{{\text{b}}} ^{\prime}/{\text{k}}_{{\text{b}}}$ = 10 (**f)**. With a larger ${\text{k}}_{{\text{b}}} ^{\prime}/{\text{k}}_{{\text{b}}}$, populations of AFs with shorter lengths increase in the cell.

To capture the basic feature of the appearance probability of actin molecules $p_{{\text{i}}}$ derived as a function of length $l_{{\text{i}}}$, the effect of changing the ratio of ${\text{k}}_{{\text{b}}}^{^{\prime}}$ to the constant ${\text{k}}_{{\text{b}}}$ was analyzed. At ${\text{k}}_{{\text{b}}}^{^{\prime}} /{\text{k}}_{{\text{b}}}$ = 1, the distribution was approximately comparable in number for all AF lengths (Fig. 2a). As ${\text{k}}_{{\text{b}}}^{^{\prime}} /{\text{k}}_{{\text{b}}}$ is raised up to 10, the distribution is skewed toward short AFs (Fig. 2b–f). Further smaller and larger ${\text{k}}_{{\text{b}}}^{^{\prime}} /{\text{k}}_{{\text{b}}}$ resulted in a distribution similar to that at ${\text{k}}_{{\text{b}}}^{^{\prime}} /{\text{k}}_{{\text{b}}}$ = 1 and 10, respectively (Supporting Material Fig. S2). Thus, lowering the relative contribution of ${\text{k}}_{{\text{b}}}^{^{\prime}}$, or namely weighting the effect of $S_{{\text{f}}}$, gives rise to a uniform distribution of AF length. Meanwhile, increasing the relative contribution of ${\text{k}}_{{\text{b}}}^{^{\prime}}$, or weighting the effect of $S_{{\text{d}}}$, gives rise to a distribution skewed to short AFs. Their log–log plots suggest that, with the raised ${\text{k}}_{{\text{b}}}^{^{\prime}} /{\text{k}}_{{\text{b}}}$, the distribution tends to approach an exponential distribution (Supporting Material Fig. S3). Thus, the overall distribution does not display a peak at a specific length as in a normal distribution and is not preferentially dominated by long AFs.

### The effect of force in AFs

**Figure 3:**
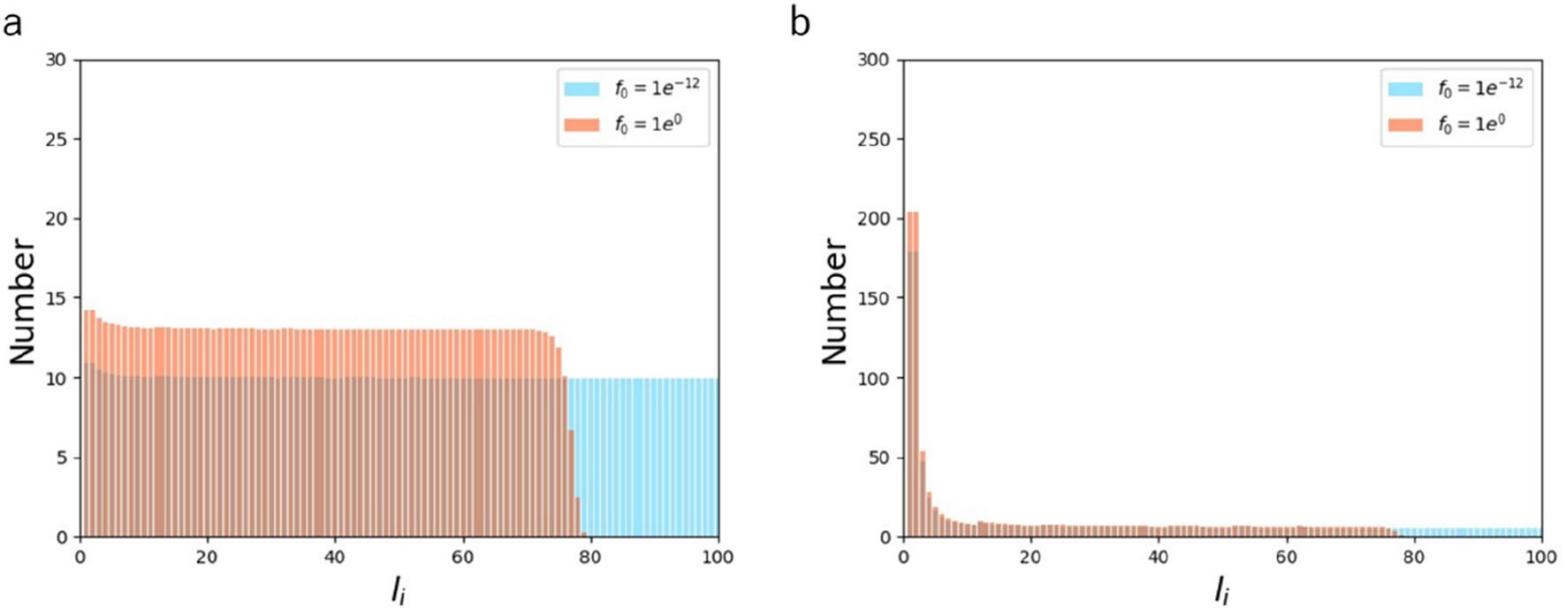
Length distribution of AFs comprising of ${\text{N}}$ = 1000 monomers with $f_{0}$ = 10^–12^ (blue) and $f_{0}$ = 1 (orange) at ${\text{k}}_{{\text{b}}} ^{\prime}/{\text{k}}_{{\text{b}}}$ = 0.1 (**a**) and ${\text{k}}_{{\text{b}}} ^{\prime}/{\text{k}}_{{\text{b}}}$ = 4 (**b**).

**Figure 4:**
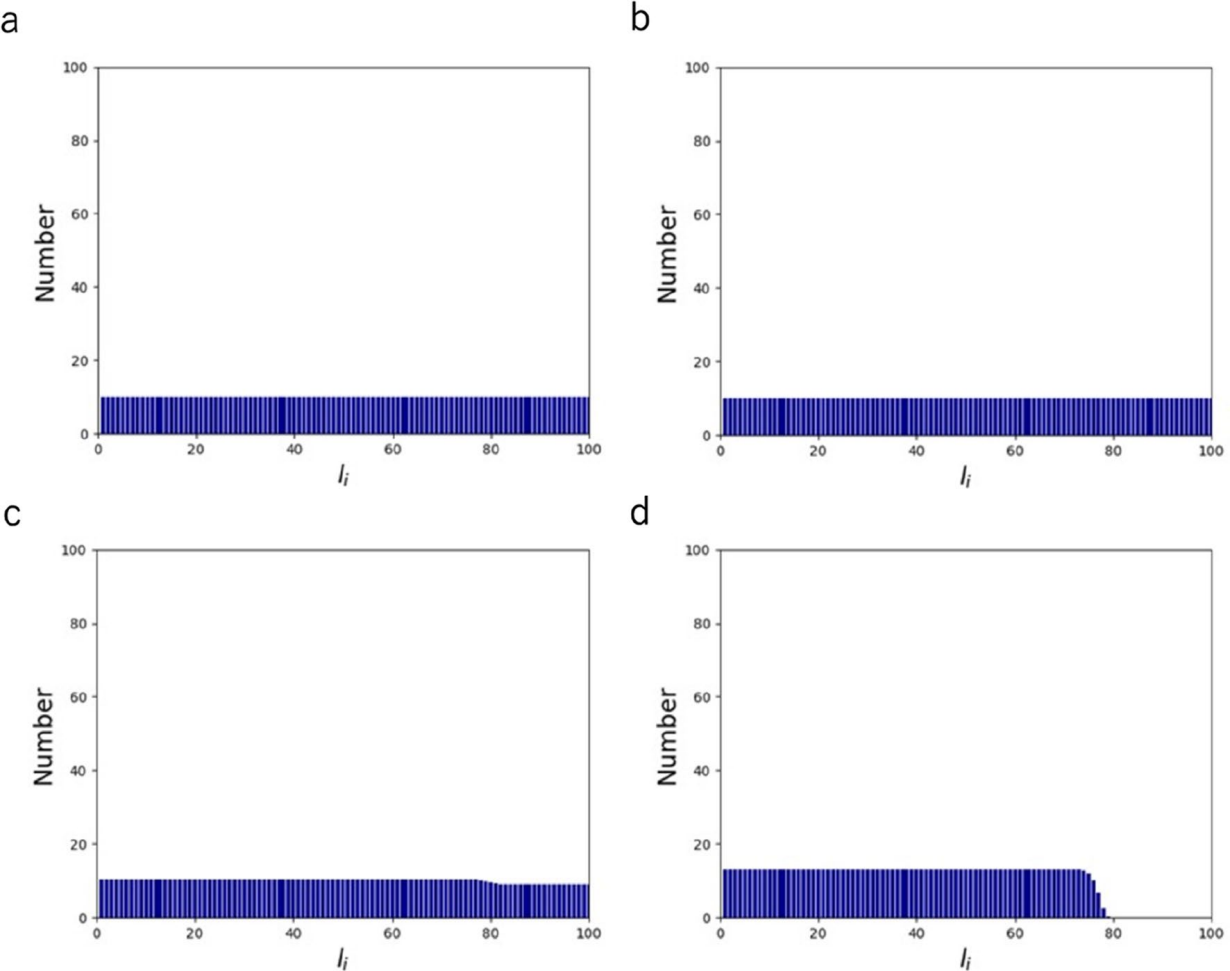
Length distribution of AFs comprising of ${\text{N}}$ = 1000 monomers at $f_{0}$ = 10^–6^ (**a**), $f_{0}$ = 10^–4^ (**b**), $f_{0}$ = 10^–2^ (**c**), and $f_{0}$ = 10^0^ (**d**).

The effect of increasing the maximum force $f_{0}$ that individual AFs sustain was analyzed at ${\text{k}}_{{\text{b}}}^{^{\prime}} /{\text{k}}_{{\text{b}}}$ = 0.1 (Fig. 3a) and 4 (Fig. 3b), in which the distribution is uniform and skewed to short AFs, respectively, as already described above. In both cases, the increase in $f_{0}$ up to 10^0^ N resulted in suppression of AF populations with a length longer than $l_{0}$ (set to be 80 here). To compensate for the suppressed population, the appearance probability was uniformly raised in the other populations. This behavior is observed because of the constraint of the expected value of force given by Eqs. (3) and (4). More specifically, long AFs have a higher chance to be involved in bearing larger forces, and thus the constraint makes long AF populations smaller in number. We also analyzed the effect of changing the threshold length $l_{0}$ in Eq. (4) while keeping $f_{0}$ unchanged at 10^–12^ N, but almost no change was observed with $l_{0}$ = 20 and 80 both at ${\text{k}}_{{\text{b}}}^{^{\prime}} /{\text{k}}_{{\text{b}}}$ = 0.1 and 4 (Supporting Material Fig. S4). In relation to investigating the role of force, the effect of changing the strain ${\uplambda }$, which was introduced in the process of defining the internal energy of the cell from a thermodynamic perspective (see Supporting Material), was also analyzed. The distribution was almost insensitive to the value of ${\uplambda }$ (0.2 or 0.8) as analyzed at ${\text{k}}_{{\text{b}}}^{^{\prime}} /{\text{k}}_{{\text{b}}}$ = 4 (Supporting Material Fig. S5). The absence of response to these parameters is touched on again in the next section.

### The individual effect of formation entropy

We have so far considered both $S_{{\text{f}}}$ and $S_{{\text{d}}}$ for characterizing the overall objective function Eq. (11). To evaluate the sole effect of $S_{{\text{f}}}$, here we put ${\text{k}}_{{\text{b}}}^{^{\prime}}$ = 0 in the probability function of Eq. (13) where the effect of $S_{{\text{d}}}$ is omitted. Unlike the case with the two entropies where short AFs appear more frequently (Fig. 2), the distribution becomes uniform all along the length at a maximum force $f_{0}$ of more than 10^–4^ N (Fig. 4a,b). As $f_{0}$ is increased to 10^–2^ N, a stepwise increase toward shorter AFs appears at the position of $l_{0}$ set here to be 80 (Fig. 4c). With a further increase in $f_{0}$ to 10^0^ N, the AF population with a length longer than $l_{0}$ completely disappears, and accordingly the short population is lifted upward (Fig. 4d). This behavior creating a threshold for appearance is consistent with the case involving the two entropies (Fig. 3) and can thus be explained in the same way as described above; namely, the increase in the maximum force lowers the appearance probability of long AF populations.

**Figure 5:**
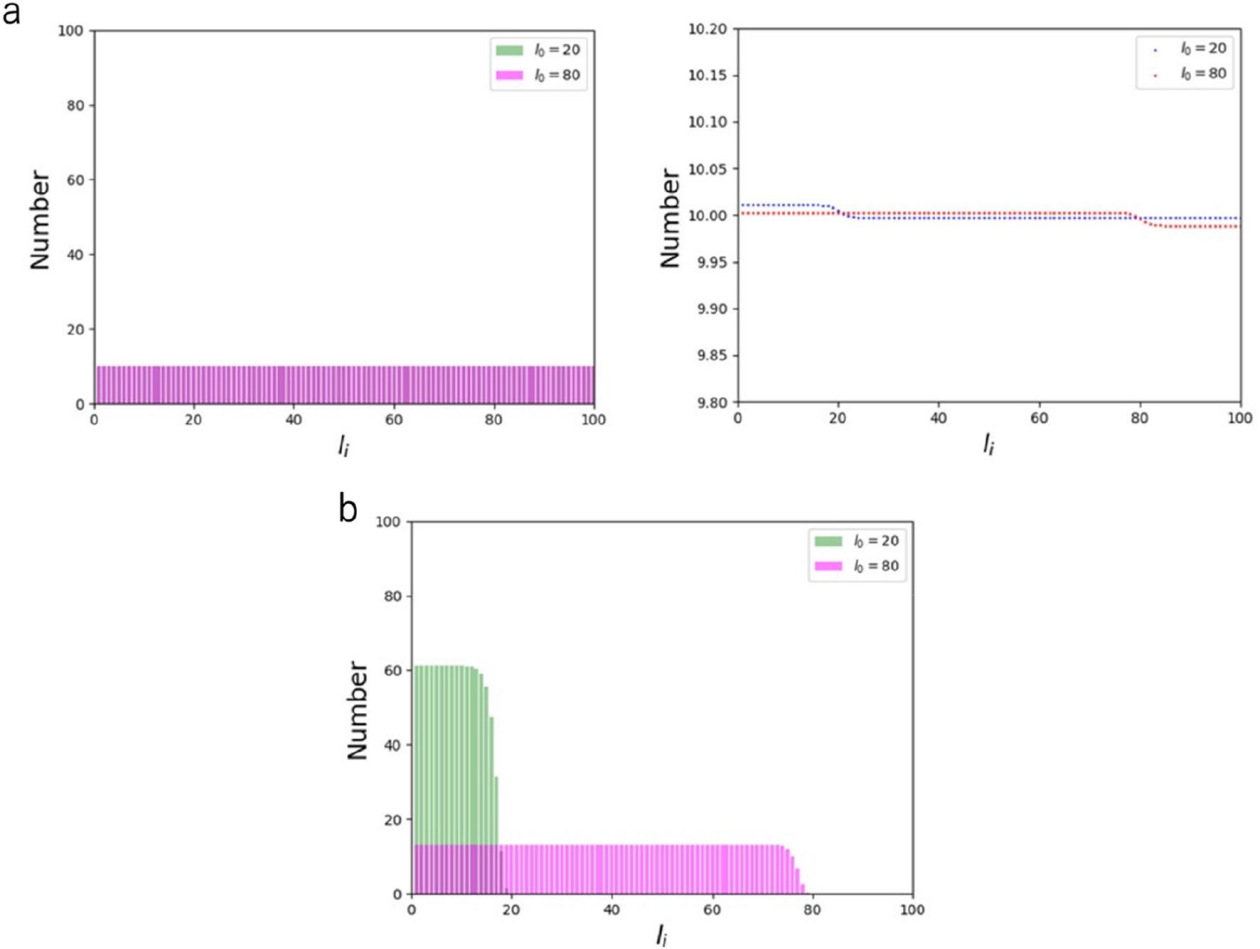
Length distribution of AFs comprising of ${\text{N}}$ = 1000 monomers with $l_{0}$ = 20 (green) and $l_{0}$ = 80 (pink) at $f_{0}$ = 10^–4^ (**a**) and $f_{0}$ = 10^0^ (**b**). For visual clarity, point plots are also shown on the right for the case of **a** with magnified vertical axis.

We next analyzed the effect of changing the threshold length $l_{0}$, which had little effect in the presence of $S_{{\text{d}}}$ in the objective function (Supporting Material Fig. S4). We found that, in the absence of $S_{{\text{d}}}$, only a subtle change is observed at a low $f_{0}$ of 10^–4^ N regardless of $l_{0}$ = 20 or 80 (Fig. 5a), while the extent of the change becomes profound at an $f_{0}$ of 10^0^ N where a steep stepwise change occurs at the position of $l_{0}$ (Fig. 5b). Nevertheless, the shape of the uniform distribution does not change except for the position of the threshold for appearance.

In relation to investigating the role of force, we analyzed the effect of ${\uplambda }$ as it partly determines the sensitivity of entropy to force, in which an increased ${\uplambda }$ is supposed to increase the sensitivity (Supporting Material Eq. S28). Unlike the case with the two entropies where ${\uplambda }$ has only little effect (Supporting Material Fig. S5), increasing ${\uplambda }$ from 0.2 to 0.8 does provide a uniform increase in the probability for AF populations with a length shorter than $l_{0}$ (Supporting Material Fig. S6). As the increase in ${\uplambda }$ strengthens the sensitivity to force, it also causes an effect similar to that caused by the increase in $f_{0}$, i.e., the uniform lift in probability below $l_{0}$ due to the constraint of constant expected value of force (Figs. 3, 4, and 5). The lift becomes particularly detectable in the absence of $S_{{\text{d}}}$ as the probability is low and flat over lengths less than $l_{0}$, while it is hard to detect the subtle change in the presence of $S_{{\text{d}}}$ where the probability is high at short lengths (Fig. S5).

### The individual effect of dispersion entropy

Unlike the above case that $S_{{\text{d}}}$ is eliminated with ${\text{k}}_{{\text{b}}}^{^{\prime}}$ = 0, $p_{{\text{i}}}$ is not obtained for the case eliminating $S_{{\text{f}}}$ as ${\text{k}}_{{\text{b}}}$ is included in the denominator of Eq. (13). As $S_{{\text{d}}}$ takes a larger value for shorter AF lengths in a monotonically varying manner (Eq. (10)), the absence of local maxima in the case considering $S_{{\text{f}}}$ as the only objective function leads to that the probability is not analytically derived by the method of Lagrange multipliers. The probability that maximizes $S_{{\text{f}}}$ under the two constraints dictating the constant amount of actin monomers and force would be one in which shorter AFs more frequently appear. Indeed, increasing the ratio of ${\text{k}}_{{\text{b}}}^{^{\prime}} /{\text{k}}_{{\text{b}}}$ (i.e., decreasing the relative impact of ${\text{k}}_{{\text{b}}}$ on ${\text{k}}_{{\text{b}}}^{^{\prime}}$) approaches such a distribution (Fig. 2).

In relation to $S_{{\text{d}}}$, the effect of $A_{{\text{i}}}$ denoting the coefficient of $n_{{\text{i}}} /l_{{\text{i}}}$ in Eq. (9) and measuring how each length population is spatially dispersed was analyzed. The relationship between $A_{{\text{i}}}$ and $l_{{\text{i}}}$ as a function of the compartment size ${\text{a}}$ shows that a smaller value of ${\text{a}}$, which more finely divides the cell, increases more steeply with $l_{{\text{i}}}$ (Supporting Material Fig. S7a). At a small ${\text{a}}$, the number of compartments occupied by AFs increases relatively sensitively with increase in AF length; meanwhile, it does not necessarily increase at a large ${\text{a}}$ until the AF length exceeds a certain level to finally make incremental changes. The length required for the stepwise increase is determined by the number of summation operations for ${\text{i}}$ in Eq. (8), specifically $\left( {1 + {\text{aj}}} \right) - \left\{ {2 + {\text{a}}\left( {{\text{j}} - 1} \right)} \right\} + 1 = {\text{a}}$; thus, each increment occurs upon a length of ${\text{a}}$. To evaluate the contribution of each actin molecule to the dispersion with different AF lengths, the number of occupied compartments per unit length (per bound actin molecule), i.e., $A_{{\text{i}}} /l_{{\text{i}}}$, was analyzed (Supporting Material Fig. S7b). $A_{{\text{i}}} /l_{{\text{i}}}$ is large for short AFs and decreases with increased AF length to converge to the reciprocal of ${\text{a}}$, indicating that the efficiency for intracellular dispersion of individual actin monomers is lowered by being associated with long AFs, but there is a limitation value determined by the compartment size ${\text{a}}$ or equivalently the monomer size. We also analyzed how different values of ${\text{a}}$ affect the length distribution of AFs at different ${\text{k}}_{{\text{b}}}^{^{\prime}} /{\text{k}}_{{\text{b}}}$ (Figs. S8 and S9). The results show that increasing ${\text{a}}$ as well as ${\text{k}}_{{\text{b}}}^{^{\prime}} /{\text{k}}_{{\text{b}}}$ both shifts the distribution toward shorter AF length populations because the increase in ${\text{a}}$ and ${\text{k}}_{{\text{b}}}^{^{\prime}} /{\text{k}}_{{\text{b}}}$ both decreases the efficiency for dispersion (Supporting Material Fig. S7) and the weight on $S_{{\text{f}}}$ that leads to a uniform distribution (Fig. 2), respectively. It is important to note that our aim here is to capture the qualitative features of the probability distribution rather than the specific number; thus, as the trend is consistent regardless of the value of ${\text{a}}$ as examined here, we kept ${\text{a}}$ = 10 for the other parts of this study.

## Discussion

Nonmuscle cells such as endothelial cells are known to adapt to mechanical stress applied from arbitrary directions^8,11,22^. What makes this mechanical adaptation possible is the presence of tensional homeostasis, which allows the cells to sustain a constant level of tension over the cytoplasm through turnover of their constituent proteins^24–26^. More specifically, well-spread cells are known to sustain a homeostatic tension, and even if mechanical perturbations are given to the cells, the level of the tension returns to the basal level at the steady states^5–13^. The set-point tension can actually be changed as a function of loading rate in fibroblasts^3^, but here we focused on stationary cells with no externally applied mechanical stress. To then address the physical requirement for the tensional homeostasis, we considered what type of actin molecule populations is required to provide a specific mean tension throughout the cytoplasm. The nature of having various AF lengths in nonmuscle cells—which are supposed to behave adaptively in accordance with time-varying intracellular and extracellular milieu—is distinct from that of muscle cells—which are in contrast specialized to exhibit unidirectional contractile activity. Accordingly, for example, nebulin is expressed specifically within muscle cells to stabilize AFs to possess a fixed length^27^.

Physicochemical mechanisms determining the length of AFs have been analyzed mostly on their in vitro dynamic behavior where typically the rate of polymerization/depolymerization, viscosity, and diffusion as a function of the length were considered^28–31^. AFs in vitro were then predicted to have an exponential distribution, which is qualitatively consistent with experimental observations^32^. The effect of some actin-binding proteins was also analyzed, yielding in some cases a distribution with a local maximum^33–38^. Regarding the AF distribution in living cells, on the other hand, its involvement in constructing filopodia/lamellipodia was investigated^39^. However, previous studies have not explicitly focused on actin meshwork, namely the basic intracellular architecture^17–20^. Thus, to our knowledge, there is no study that investigated how individual cells are endowed with tensional homeostasis thanks to their AF resources. Unlike in vitro studies, there are only limited experimental data available on the AF length distribution within animal cells, apart from that in Dictyostelium discoideum^40^; but, observations of cell breakage after phalloidin-mediated AF stabilization suggest that the probability is clearly higher for shorter AFs within cells^41^, thus consistent with our prediction.

To extract the physical features of tensional homeostasis, we considered the two independent factors: $S_{{\text{f}}}$ and $S_{{\text{d}}}$ that are related to the formation of the force-bearing elements and to the spatial distribution of the elements, respectively. $S_{{\text{f}}}$ indicates in what manner each AF is likely to be formed. For example, if all the molecules have the same length, the number of states is then unity, meaning that they can only be in a fixed state and are thus unstable as a whole since stochastic fluctuations are not allowed. If actin molecules instead take various lengths, they would be more stabilized by having a larger number of states. $S_{{\text{d}}}$, on the other hand, indicates in what manner AFs are likely to be dispersed in the cell. Because these two quantities are both identical in concept to entropy, we introduced ${\text{k}}_{{\text{b}}}^{^{\prime}}$ to modulate the weight of $S_{{\text{d}}}$ versus $S_{{\text{f}}}$, enabling the comparison of the relative impact to the overall system. With regards to the importance of $S_{{\text{d}}}$, recent studies have demonstrated that mechanosensitive actin-binding proteins such as filamin are distributed over the cytoplasm^22,42,43^. These cytoplasmic sensors play a role in maintaining cell functions by converting mechanical information such as intracellular tension into biochemical information such as change in binding affinity, presumably locally detecting deviation in the homeostatic tension and hence keeping the functional integrity of the cell^14^. The intracellular dispersion of AFs may thus not only be induced according to the natural law of physics, but also it may be serving for such biological functions.

Studies aimed at characterizing the properties of individual proteins have been extensively conducted as technical advances have made it possible to perform sophisticated measurements at the molecular level, while their collective behaviors are not necessarily taken into sufficient account. However, living organisms are not merely a collection of elements with separate functions but exist through complex interactions among structurally and functionally hierarchical “layers”, thereby allowing the whole system to acquire universal features of life such as flexibility, adaptivity, and resulting maintenance of biological activities. It is necessary, given the actual complexity of cells, to limit the scope of our research to arguably the most essential factor in tensional homeostasis, i.e., the involvement of AFs; but, by describing a simple model, we captured the salient features at the cellular and molecular levels. Specifically, we derived the length distribution of AFs not by considering isolated individuals but by analyzing the population, in which the cellular constraints of having a constant level of actin monomers as well as tension turn out that the probability decreases as the length increases. Our prediction is in agreement with experimental observations made on cells^41^. Exploring the involvement of other actin-regulating proteins^44^ will be the subject of future challenging but exciting investigations, for which our approach would provide a basis. To this end, as we discussed just after the introduction of the overall entropy of Eq. (11), further elaborate formulations of entropies and appropriate constraints are key to describing the characteristics and roles of the newly added elements.

## Supplementary Information

Supplementary Information.

## Data Availability

All data generated or analyzed during this study are included in this published article and its supplementary information files.
