# Supplementary material for: A statistical mechanics model for determining the length distribution of actin filaments under cellular tensional homeostasis
Source: Sci Rep. 2022 Aug 24;12:14466. doi: 10.1038/s41598-022-18833-1 (PMC9402564; doi:10.1038/s41598-022-18833-1)
Supplement: Supplementary file 1 — Supplementary Information. [file 41598_2022_18833_MOESM1_ESM.pdf]

Supporting Material for Ueda et al. “A statistical mechanics model for determining the length distribution of actin filaments under cellular tensional homeostasis”

Contents:

S1: Figure S1

S2: Derivation of Equation (7)

S3: Explanation of Equation (8)

S4: Derivation of Equation (13)

S5: Figure S2

S6: Figure S3

S7: Figure S4

S8: Figure S5

S9: Figure S6

S10: Figure S7

S11: Figure S8

S12: Figure S9

S1: Figure S1

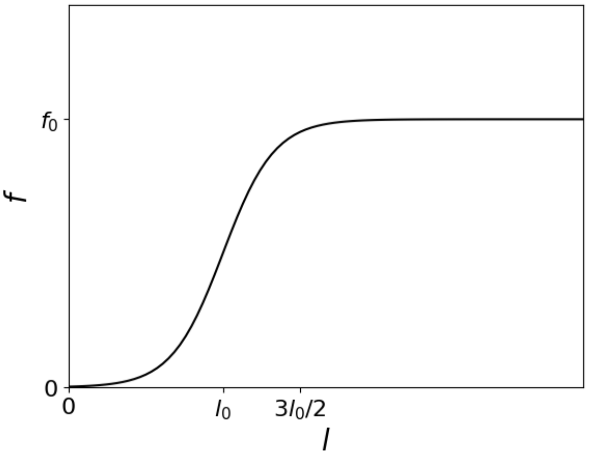

Fig. S1 The relationship between force f_{i} and AF length l_{i}. The force increases up to f_0, where l_0 characterizes the position of the transition.

S2: Derivation of Eq. (7)

From Eqs. (5) and (6), the formation-associated entropy is described by

$$S_\mathrm{f} = \mathrm{k_b} \ln \frac{\mathrm{N}!}{n_1!\, n_2! \cdots n_\mathrm{m}!} \tag{S1}$$

Stirling's approximation on Eq. (S1) together with Eq. (1) yields that

$$\begin{aligned} S_\mathrm{f} &= \mathrm{k_b}\big(\ln \mathrm{N}! - (\ln n_1! + \ln n_2! + \cdots)\big) \\ &= \mathrm{k_b}\left(\ln \mathrm{N}! - \sum \ln n_\mathrm{i}!\right) \\ &= \mathrm{k_b}\left(\mathrm{N}(\ln \mathrm{N} - 1) - \sum n_\mathrm{i}(\ln n_\mathrm{i} - 1)\right) \\ &= \mathrm{k_b}\left(\mathrm{N}\ln \mathrm{N} - \sum n_\mathrm{i} \ln n_\mathrm{i}\right). \end{aligned} \tag{S2}$$

With Eq. (2),

$$\begin{aligned} S_\mathrm{f} &= \mathrm{k_b}\left(\mathrm{N}\ln \mathrm{N} - \sum \mathrm{N} p_\mathrm{i} \ln(\mathrm{N} p_\mathrm{i})\right) \\ &= \mathrm{k_b}\left(\mathrm{N}\ln \mathrm{N} - \sum \mathrm{N} p_\mathrm{i}(\ln \mathrm{N} + \ln p_\mathrm{i})\right) \\ &= -\mathrm{k_b} \mathrm{N} \sum p_\mathrm{i} \ln p_\mathrm{i}. \end{aligned} \tag{7}$$

S3: Explanation of Eq. (8)

$$G = \frac{n_1}{l_1} + 2\sum_{\mathrm{i}=2}^{1+\mathrm{a}} \frac{n_\mathrm{i}}{l_\mathrm{i}} + 3\sum_{\mathrm{j}=2+\mathrm{a}}^{1+2\mathrm{a}} \frac{n_\mathrm{i}}{l_\mathrm{i}} + \cdots = \frac{n_1}{l_1} + \sum_{\mathrm{j}=1}^{\frac{m-2}{\mathrm{a}}+1} \sum_{\mathrm{i}=2+\mathrm{a}(\mathrm{j}-1)}^{1+\mathrm{aj}} (\mathrm{j}+1)\frac{n_\mathrm{i}}{l_\mathrm{i}} \qquad (8)$$

As described in the main text, n_i is the number of actin monomers in the cell involved in constructing AFs of length l_i. Thus, $n_\mathrm{i}/l_\mathrm{i}$ represents the number of individual AFs of length l_i. The diameter of each molecule is smaller than a that is the compartment size, indicating that the number of compartments that can be occupied by a single actin monomer (or an AF of length l_1) is always unity as the monomer is the smallest countable unit, which determines the first term in the middle expression of Eq. (8). Thus, i = 1 represents a group of AFs with the shortest length. As described in the main text, the diameter and filament lengths of actin molecules are normalized by the unit length l_1 = 1. The compartment size of a = 1 means that the diameter of actin molecules is equal to the length of each compartment; thus, the number of compartments occupied by a single AF with a length of, e.g., l_i = 10 is also 10. Let us consider how the occupied compartments are counted in general with a > 1 based on some examples shown in Fig. 1b where a case with $\mathrm{a} = 3l_1 = 3$ is drawn. Here, the maximum number of compartments that can be occupied by two (l_2), three (l_3), and four (l_4) bound actin molecules is all 2 as indicated by the gray-shaded area in the figure. If actin polymerizes to have a length of l_5 composed of five actin monomers, the maximum number of compartments occupied by this AF is 3 as again indicated by the gray-shaded area. The count for these 2 and 3 consecutive compartments corresponds to the second and third terms in the middle expression of Eq. (8), respectively. Here, the number of the AFs is also considered; namely, in the case of the population of i = 5, the number of the AFs is n_5/l_5 = 15/5 = 3. The general form, in which the maximum number of the compartments occupied by AFs is counted in the same way, corresponds to the right expression of Eq. (8), where m is, again, a group of AFs with the longest length.

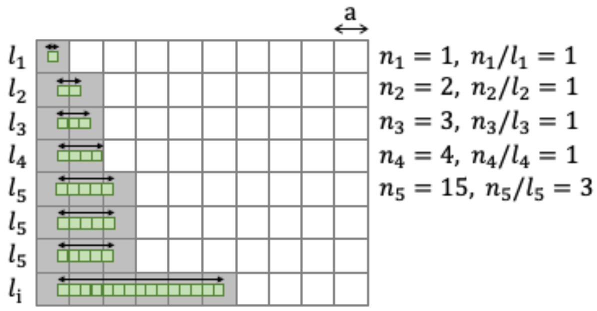

Fig. 1b

S4: Derivation of Eq. (14)

The partial derivative of Eq. (13) with respect to p_{i} is

$$\frac{\partial \mathcal{L}}{\partial p_{\mathrm{i}}}=\sum\left[-\mathrm{k_b N ln}p_{\mathrm{i}}-\mathrm{k_b N}+\mathrm{k'_b N}\frac{A_{\mathrm{i}}}{l_{\mathrm{i}}}-\alpha f_{\mathrm{i}}-\beta\right]. \tag{S3}$$

The objective function $\mathcal{L}$ takes an extreme value at

$$\frac{\partial \mathcal{L}}{\partial p_{\mathrm{i}}}=0, \tag{S4}$$

which provides the existence probability for the present purpose

$$p_{\mathrm{i}}=\exp\left[\left(\mathrm{k'_b}\frac{A_{\mathrm{i}}}{l_{\mathrm{i}}}-\frac{\alpha}{\mathrm{N}}f_{\mathrm{i}}-\frac{\beta}{\mathrm{N}}\right)/\mathrm{k_b}-1\right]. \tag{S5}$$

Next, to determine the Lagrange multipliers α and β, Eq. (S5) is substituted partially into Eq. (11) for the overall entropy, yielding

$$S=-\mathrm{k_b N}\sum p_{\mathrm{i}}\left[\left(\mathrm{k'_b}\frac{A_{\mathrm{i}}}{l_{\mathrm{i}}}-\frac{\alpha}{\mathrm{N}}f_{\mathrm{i}}-\frac{\beta}{\mathrm{N}}\right)/\mathrm{k_b}-1\right]+\mathrm{k'_b}\sum \mathrm{N}\frac{A_{\mathrm{i}}}{l_{\mathrm{i}}}p_{\mathrm{i}}$$

$$=\alpha\sum p_{\mathrm{i}}f_{\mathrm{i}}+\beta\sum p_{\mathrm{i}}-\mathrm{k_b N}. \tag{S6}$$

With Eqs. (1)–(3), Eq. (S6) is expressed as

$$S=\alpha F+\beta-\mathrm{k_b N}. \tag{S7}$$

The partial derivative of Eq. (S7) with respect to the expected value of force F is

$$\frac{\partial S}{\partial F}=\alpha+F\frac{\partial \alpha}{\partial F}+\frac{\partial \beta}{\partial F}. \tag{S8}$$

With Eqs. (1) and (2), the summation of p_{i} shown by Eq. (S5) must be unity:

$$\sum\exp\left[\left(\mathrm{k'_b}\frac{A_{\mathrm{i}}}{l_{\mathrm{i}}}-\frac{\alpha}{\mathrm{N}}f_{\mathrm{i}}-\frac{\beta}{\mathrm{N}}\right)/\mathrm{k_b}-1\right]=1. \tag{S9}$$

The partial derivative of Eq. (S9) with respect to F is

$$\sum\frac{1}{\mathrm{k_b}}\left(-\frac{f_{\mathrm{i}}}{\mathrm{N}}\frac{\partial \alpha}{\partial F}-\frac{1}{\mathrm{N}}\frac{\partial \beta}{\partial F}\right)\exp\left[\left(\mathrm{k'_b}\frac{A_{\mathrm{i}}}{l_{\mathrm{i}}}-\frac{\alpha}{\mathrm{N}}f_{\mathrm{i}}-\frac{\beta}{\mathrm{N}}\right)/\mathrm{k_b}-1\right]=0. \tag{S10}$$

With Eqs. (1), (2), and (S5), Eq. (S10) is reduced to

$$-\frac{1}{\mathrm{k_b}}\left\{\sum\left(\frac{f_{\mathrm{i}}}{\mathrm{N}}\frac{\partial \alpha}{\partial F}+\frac{1}{\mathrm{N}}\frac{\partial \beta}{\partial F}\right)p_{\mathrm{i}}\right\}=0, \tag{S11}$$

and therefore

$$F\frac{\partial \alpha}{\partial F}+\frac{\partial \beta}{\partial F}=0. \tag{S12}$$

With Eq. (S8), the partial derivative of the overall entropy S with respect to F is

$$\frac{\partial S}{\partial F}=\alpha, \tag{S13}$$

suggesting that the Lagrange multiplier introduced to find the optimal distribution, α, can be expressed using the overall entropy and force expectation value.

Next, we discuss the specific form of α given that the entropy derived from a microscopic point of view based on statistical mechanics, maximizing the objective function at equilibrium, coincides with that derived from a macroscopic point of view based on thermodynamics. For the thermodynamic consideration, we consider a cell with a volume of V and a preexisting strain of λ. As we already discussed, the presence of the preexisting tension (or concomitant mechanical strain) is aimed at capturing the feature of tensional homeostasis. The strain varies spatially and temporarily at the microscopic view, while at the macroscopic view we consider an average level of strain distributed throughout the cytoplasm. The expected value of force F introduced in Eq. (3) is regarded as stress in the following thermodynamic model.

The first law of thermodynamics gives

$$\mathrm{d}U = \mathrm{d}'Q + \mathrm{d}'W \tag{S14}$$

where U, Q, and W denote the internal energy, the heat given to the system, and the work done to the system, respectively. Let us assume an elastic relationship between stress F and strain λ, i.e.,

$$F = \mathrm{E}\lambda \tag{S15}$$

where E denotes the elastic modulus of the cell, and it turns out that the strain energy is

$$k = \int F\mathrm{d}\lambda = \frac{1}{2}\mathrm{E}\lambda^2 = \frac{1}{2}F\lambda, \tag{S16}$$

and accordingly the whole strain energy is described using volume V to be

$$K = \mathrm{V}k = \frac{1}{2}\mathrm{V}F\lambda. \tag{S17}$$

The work caused by change in stress, which in other words is the change in strain energy, is therefore

$$\mathrm{d}'W_{\Delta\mathrm{stress}} = \frac{1}{2}\mathrm{V}\lambda\mathrm{d}F, \tag{S18}$$

in which cell volume is assumed to be constant (i.e., Poisson's ratio = 0.5).

The strain or intracellular deformation is generated within cells at tensional homeostasis upon the actin–myosin II interaction using the energy obtained from the ATP-mediated chemical reaction. Therefore, the strain energy discussed here is not the result of work done "by external forces" as in the case of conventional spring elasticity, but is the result of work done "by the system itself." In other words, as the cell system we discuss here includes subcellular parts that do work (namely, generate force and deform), the work done to the system must be opposite in sign to that done "by the system itself." It also turns out that, in

the present system, the work done "by the system itself" will increase in amount as the strain/deformation-associated intracellular stress F increases. Therefore, change in work of the entire system is

$$\mathrm{d}'W = -\mathrm{d}'W_{\Delta\mathrm{stress}} = -\frac{1}{2}\mathrm{V}\lambda\mathrm{d}F. \tag{S19}$$

With this, Eq. (S14) turns out

$$\mathrm{d}U = T\mathrm{d}S - \frac{1}{2}\mathrm{V}\lambda\mathrm{d}F \tag{S20}$$

where T is the thermodynamic temperature. The free energy G is defined as

$$G = U - TS, \tag{S21}$$

and its change is

$$\mathrm{d}G = \mathrm{d}U - \mathrm{Td}S - S\mathrm{d}T, \tag{S22}$$

or with Eq. (S20)

$$\mathrm{d}G = -\frac{1}{2}\mathrm{V}\lambda\mathrm{d}F - S\mathrm{d}T. \tag{S23}$$

Describing the total differential form of $G(F,T)$,

$$\mathrm{d}G(F,T) = \left(\frac{\partial G}{\partial F}\right)_T \mathrm{d}F + \left(\frac{\partial G}{\partial T}\right)_F \mathrm{d}T. \tag{S24}$$

From Eqs. (S23) and (S24),

$$\left(\frac{\partial G}{\partial F}\right)_T = -\frac{1}{2}\mathrm{V}\lambda. \tag{S25}$$

The partial derivative of the free energy with respect to F at constant temperature yields

$$\left(\frac{\partial G}{\partial F}\right)_T = \left(\frac{\partial U}{\partial F}\right)_T - T\left(\frac{\partial S}{\partial F}\right)_T. \tag{S26}$$

From Eqs. (S25) and (S26),

$$-\frac{1}{2}\mathrm{V}\lambda = \left(\frac{\partial U}{\partial F}\right)_T - T\left(\frac{\partial S}{\partial F}\right)_T. \tag{S27}$$

At constant temperature where there is no change in internal energy, Eq. (S27) is reduced to

$$\left(\frac{\partial S}{\partial F}\right)_T = \frac{\mathrm{V}\lambda}{2T}, \tag{S28}$$

describing the effect of intracellular stress on entropy derived from a macroscopic thermodynamic point of view. This positive relationship – distinct from the negative one for a conventional spring where stretch reduces the extent of fluctuations, the number of possible microstates of the constituents, and thereby the entropy as well – is reasonable because the stress of current interest F is, as already mentioned, originated from the intracellular actin–myosin II interaction. More specifically, F is interpreted to be an indicator of the activity of

the intracellular components rather than a factor compelling their movement. Thus, an increased F and resulting activation of the actin–myosin II interaction stabilize the cell structure as well as increase the entropy. Given that Eqs. (S13) and (S28) are equal at equilibrium,

$$\alpha = \frac{\mathrm{V}\lambda}{2T}. \tag{S29}$$

Substituting Eq. (S29) into (S5) yields

$$p_\mathrm{i} = \exp\left[\left(\mathrm{k}_\mathrm{b}'\frac{A_\mathrm{i}}{l_\mathrm{i}} - \frac{\mathrm{V}\lambda}{2T\mathrm{N}}f_\mathrm{i} - \frac{\beta}{\mathrm{N}}\right)/\mathrm{k}_\mathrm{b} - 1\right]. \tag{S30}$$

To determine β, the constant part of Eq. (S30), which is independent of i, was expressed as B, and then

$$p_\mathrm{i} = \mathrm{B}\exp\left[\left(\mathrm{k}_\mathrm{b}'\frac{A_\mathrm{i}}{l_\mathrm{i}} - \frac{\mathrm{V}\lambda}{2T\mathrm{N}}f_\mathrm{i}\right)/\mathrm{k}_\mathrm{b}\right] \tag{S31}$$

where

$$\mathrm{B} = \frac{1}{\exp\left[\frac{\beta}{\mathrm{k}_\mathrm{b}\mathrm{N}} + 1\right]}. \tag{S32}$$

With Eqs. (1) and (2), the summation of p_i must be unity so that

$$\sum \mathrm{B}\exp\left[\left(\mathrm{k}_\mathrm{b}'\frac{A_\mathrm{i}}{l_\mathrm{i}} - \frac{\mathrm{V}\lambda}{2T\mathrm{N}}f_\mathrm{i}\right)/\mathrm{k}_\mathrm{b}\right] = 1, \tag{S33}$$

indicating that

$$\mathrm{B} = 1/\sum \exp\left[\left(\mathrm{k}_\mathrm{b}'\frac{A_\mathrm{i}}{l_\mathrm{i}} - \frac{\mathrm{V}\lambda}{2T\mathrm{N}}f_\mathrm{i}\right)/\mathrm{k}_\mathrm{b}\right], \tag{S34}$$

and

$$\beta = -\mathrm{k}_\mathrm{b}\mathrm{N}(\ln\mathrm{B} + 1). \tag{S33}$$

Consequently,

$$p_\mathrm{i} = \frac{\exp\left[\left(\mathrm{k}_\mathrm{b}'\frac{A_\mathrm{i}}{l_\mathrm{i}} - \frac{\mathrm{V}\lambda}{2T\mathrm{N}}f_\mathrm{i}\right)/\mathrm{k}_\mathrm{b}\right]}{\sum \exp\left[\left(\mathrm{k}_\mathrm{b}'\frac{A_\mathrm{i}}{l_\mathrm{i}} - \frac{\mathrm{V}\lambda}{2T\mathrm{N}}f_\mathrm{i}\right)/\mathrm{k}_\mathrm{b}\right]}. \tag{13}$$

S5: Figure S2

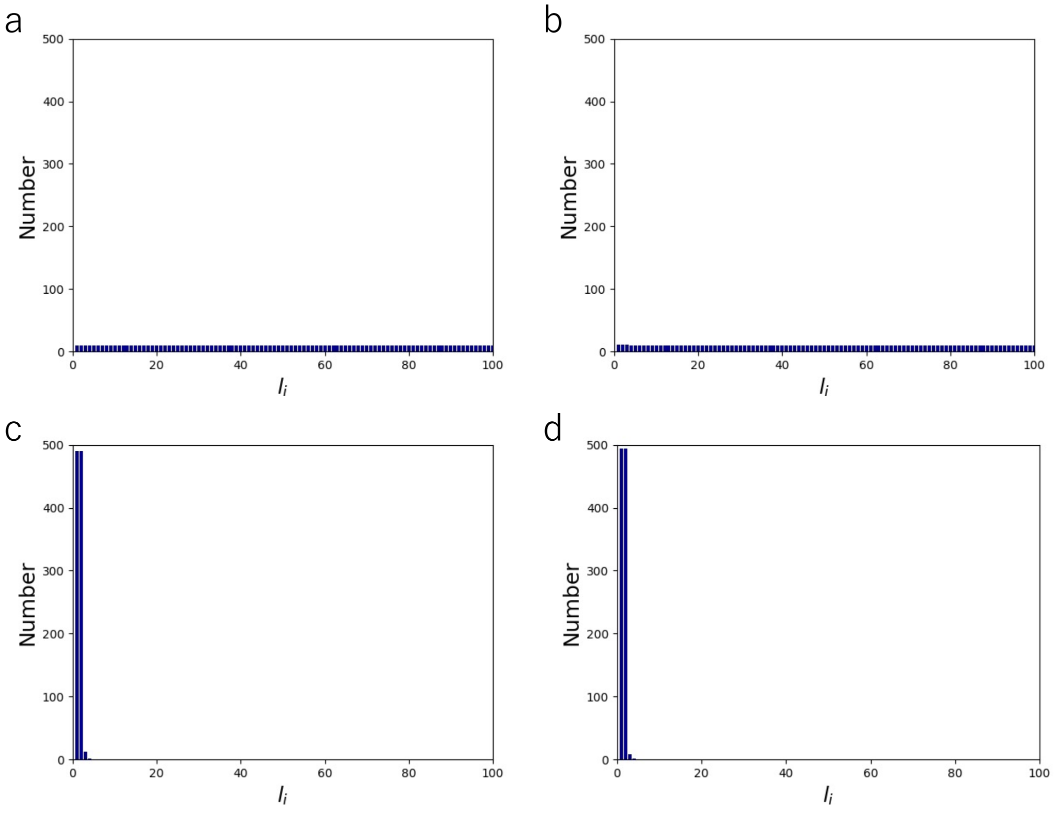

Fig. S2 Length distribution of AFs comprising of N = 1,000 monomers at k_b'/k_b = 10^{-2} (a), k_b'/k_b = 10^{-1} (b), k_b'/k_b = 11 (c), and k_b'/k_b = 12 (d).

S6: Figure S3

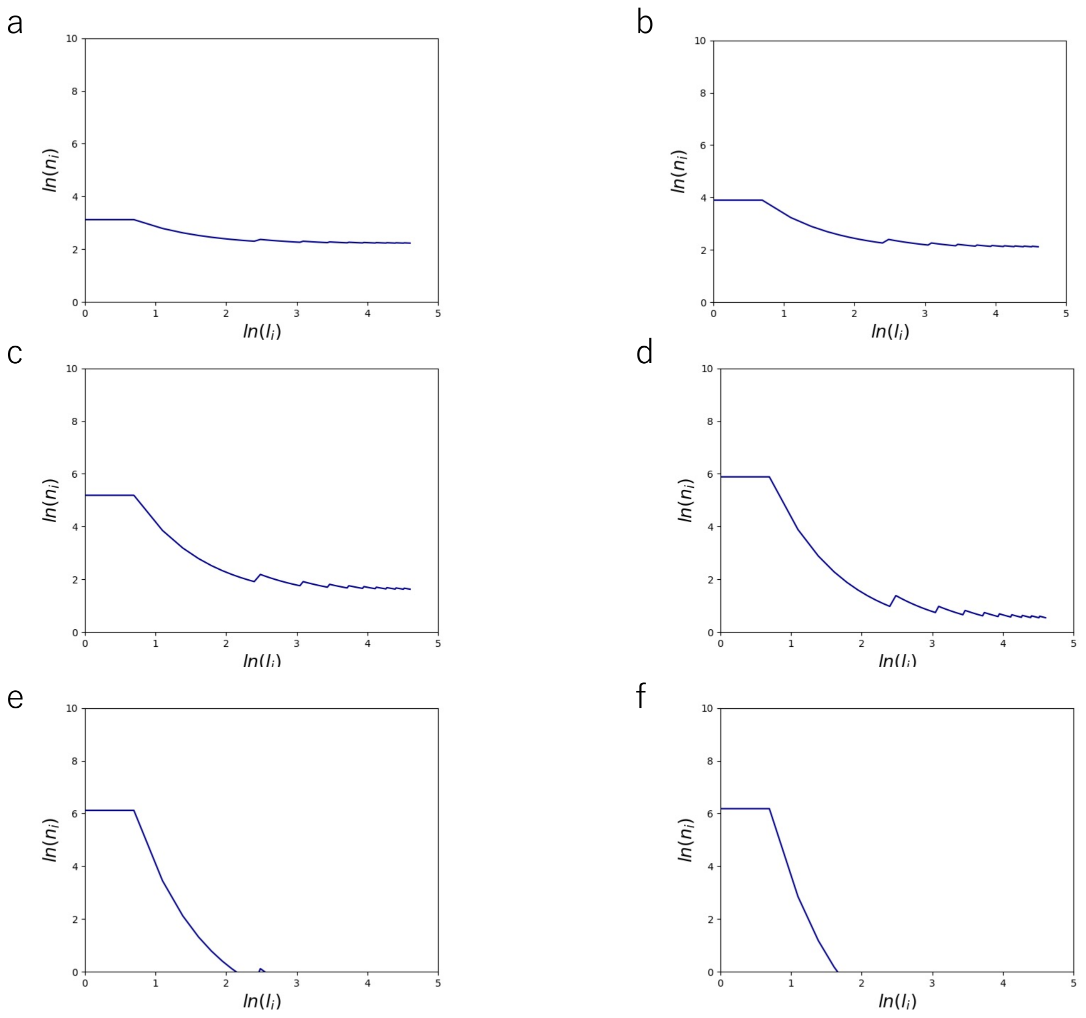

Fig. S3 Log-log graphs of Fig. 2, showing the length distribution of AFs comprising of N = 1,000 monomers at k_b'/k_b = 1 (a), k_b'/k_b = 2 (b), k_b'/k_b = 4 (c), k_b'/k_b = 6 (d), k_b'/k_b = 8 (e), and k_b'/k_b = 10 (f).

S7: Figure S4

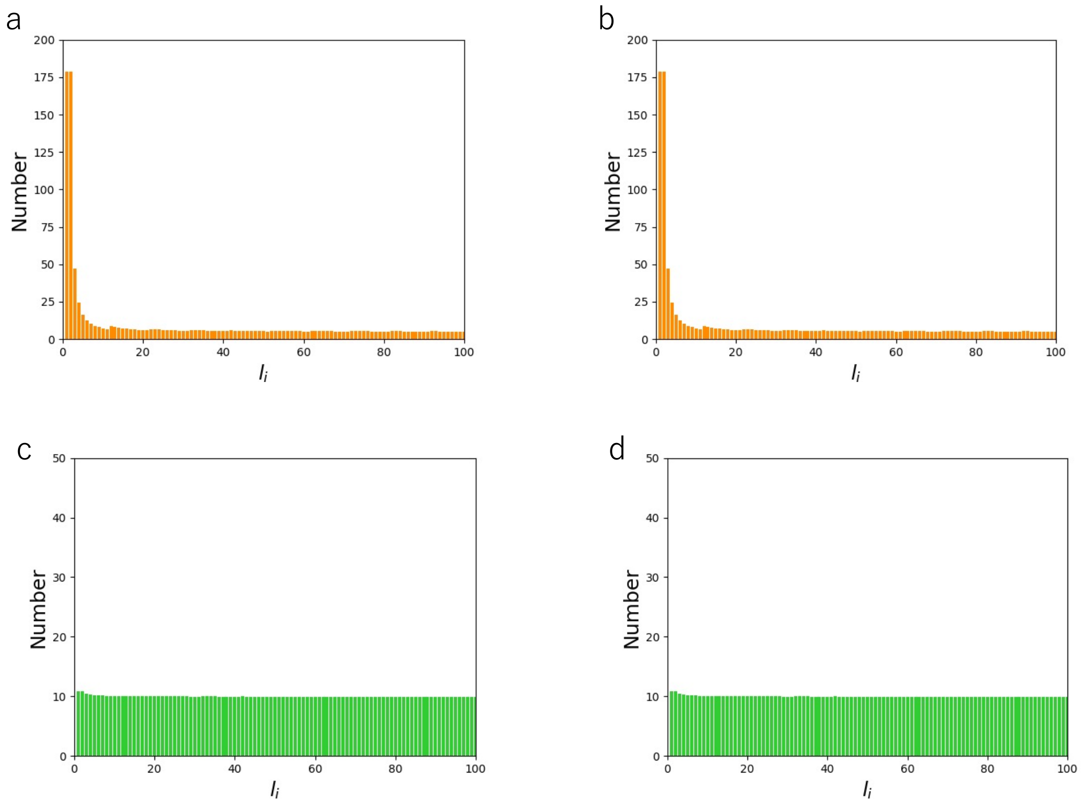

Fig. S4 Length distribution of AFs comprising of N = 1,000 monomers at $\mathrm{k_b}'/\mathrm{k_b}$ = 4 and l_0 = 20 (a), $\mathrm{k_b}'/\mathrm{k_b}$ = 4 and l_0 = 80 (b), $\mathrm{k_b}'/\mathrm{k_b}$ = 0.1 and l_0 = 20 (c), and $\mathrm{k_b}'/\mathrm{k_b}$ = 0.1 and l_0 = 80 (d), showing that the distribution does not depend on l_0.

S8: Figure S5

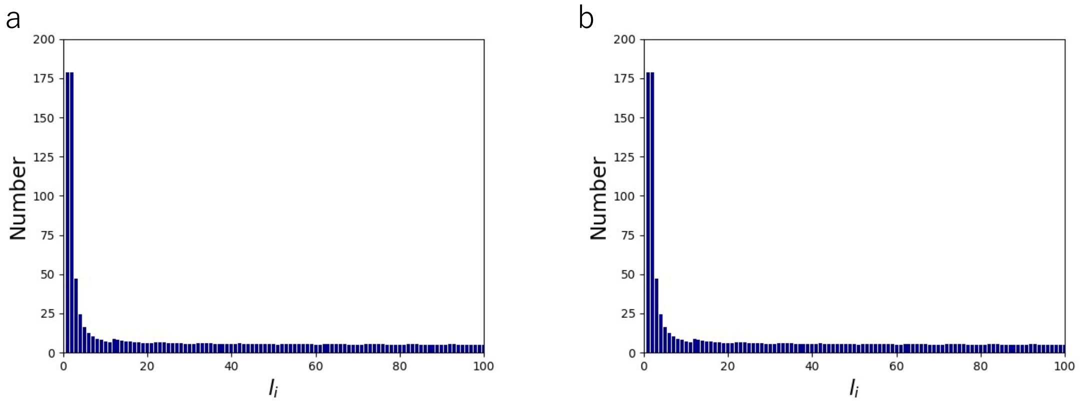

Fig. S5 Length distribution of AFs comprising of N = 1,000 monomers at preexisting cell strain (prestretch) of λ = 0.2 (a) and λ = 0.8 (b).

S9: Figure S6

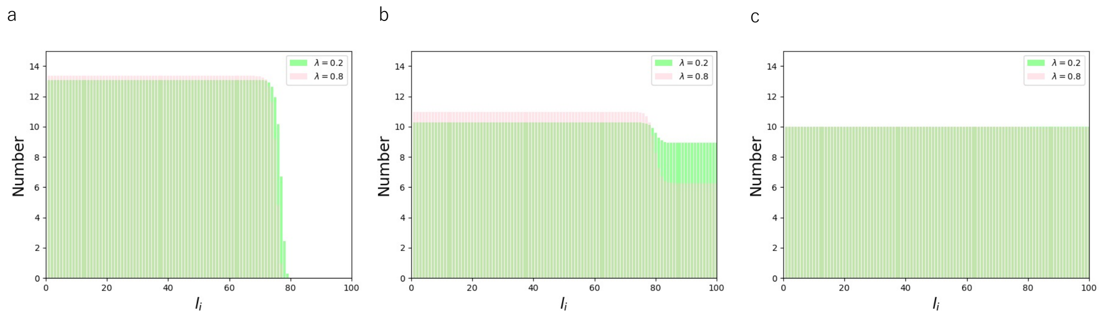

Fig. S6 Length distribution of AFs comprising of N = 1,000 monomers for preexisting cell strain of λ = 0.2 (green) and λ = 0.8 (pink) at f_0 = 10^0 (a), f_0 = 10^{-2} (b), and f_0 = 10^{-4} (c).

S10: Figure S7

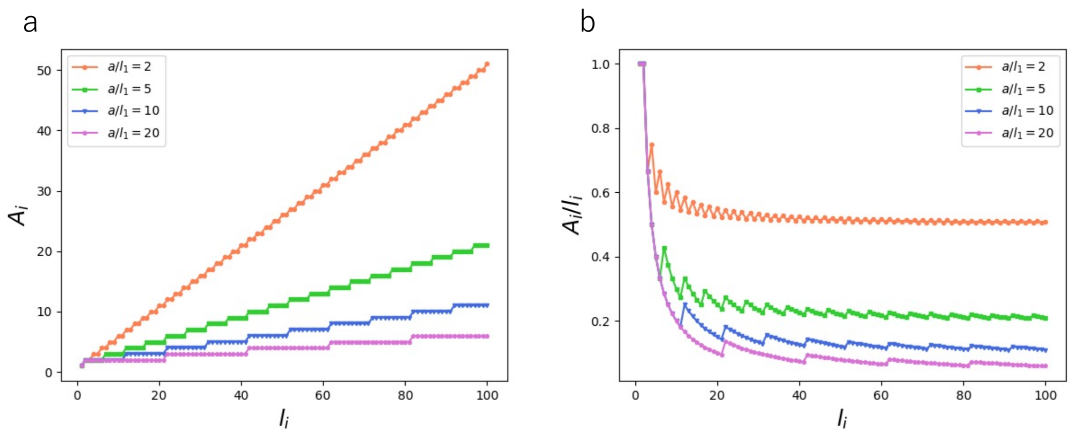

Fig. S7 The effect of changing the ratio of compartment size a to actin monomer size l_1. In the analysis, the size is normalized by l_1 as described in Methods section. (a) A_i, which determines S_d, as a function of l_1 at different a; a/l_1 = 2 (orange), $\mathrm{a}/l_1 = 5$ (green), a/l_1 = 10 (blue), and $\mathrm{a}/l_1 = 20$ (purple). (b) A_i/l_i as a function of l_1 at different a; a/l_1 = 2 (orange), a/l_1 = 5 (green), a/l_1 = 10 (blue), and a/l_1 = 20 (purple). Note that l_1 = 1 for the rest of the analyses in the present study.

S11: Figure S8

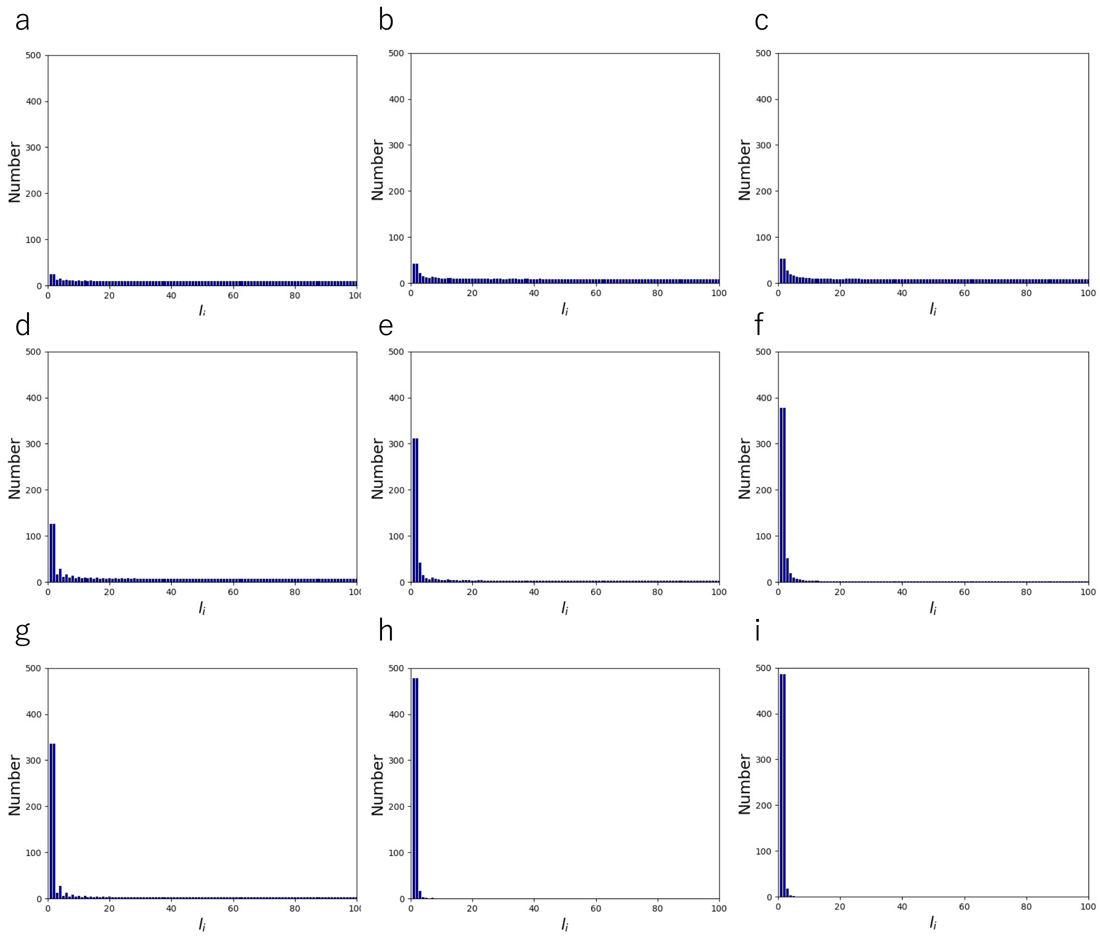

Fig. S8 Length distribution of AFs comprising of N = 1,000 monomers at $\mathrm{k_b}'/\mathrm{k_b}$ = 2 and a/l_1 = 2 (a), $\mathrm{k_b}'/\mathrm{k_b}$ = 2 and a/l_1 = 5 (b), $\mathrm{k_b}'/\mathrm{k_b}$ = 2 and a/l_1 = 20 (c), $\mathrm{k_b}'/\mathrm{k_b}$ = 6 and a/l_1 = 2 (d), $\mathrm{k_b}'/\mathrm{k_b}$ = 6 and a/l_1 = 5 (e), $\mathrm{k_b}'/\mathrm{k_b}$ = 6 and a/l_1 = 20 (f), $\mathrm{k_b}'/\mathrm{k_b}$ = 10 and a/l_1 = 2 (g), $\mathrm{k_b}'/\mathrm{k_b}$ = 10 and a/l_1 = 5 (h), and $\mathrm{k_b}'/\mathrm{k_b}$ = 10 and a/l_1 = 20 (i). Cases at a/l_1 = 10 are shown in Fig. 2.

S12: Figure S9

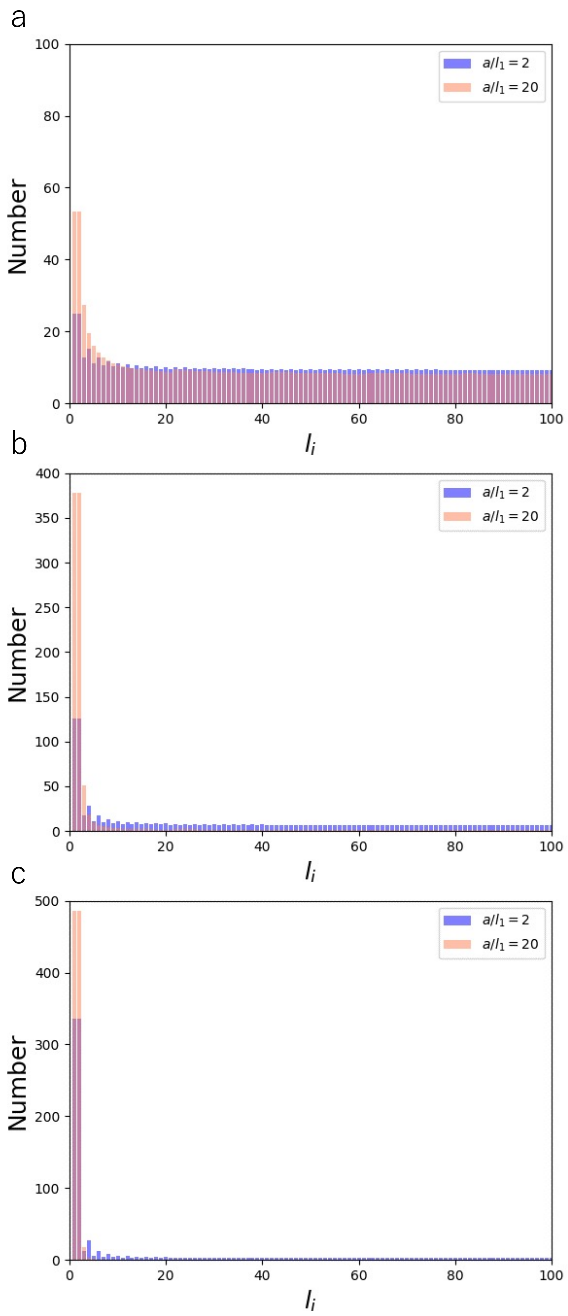

Fig. S9 Length distribution of AFs comprising of N = 1,000 monomers for a/l_1 = 2 (blue) and a/l_1 = 20 (orange) at k_b'/k_b = 2 (a), k_b'/k_b = 6 (b), and k_b'/k_b = 10 (c) taken partly from Fig. S8.
